# Disseminated *Scedosporium apiospermum* infection in a Maremmano-Abruzzese sheepdog

**DOI:** 10.1186/s12917-020-02597-9

**Published:** 2020-10-02

**Authors:** Giovanni Di Teodoro, Daniela Averaimo, Miria Primavera, Doriana Santoleri, Giorgia Giovannini, Antonio Cocco, Gabriella Di Francesco, Daniela Malatesta, Sabrina Defourny, Nicola D’Alterio, Valentina Curini, Marco Di Domenico, Antonio Petrini

**Affiliations:** 1 Istituto Zooprofilattico Sperimentale dell’Abruzzo e Molise “G. Caporale”, Campo Boario, 64100 Teramo, Italy; 2 Clinica Veterinaria Guardiese, Via Anello, 66016, Guardiagrele, Chieti, Italy

**Keywords:** Case report, Dog, Systemic mycosis, *Scedosporium apiospermum*, Diagnosis

## Abstract

**Background:**

Few cases of scedosporiosis have been reported in animals, but the true prevalence is probably underestimated due to a lack of awareness. Scedosporiosis in dogs has often been associated with localized infection (i.e., nasal infection, eumycetoma, or keratomycosis) or, in rare cases, disseminated infections.

**Case presentation:**

This case report describes the clinical and pathological features and the diagnostic process of a rare systemic and fatal fungal infection in a dog caused by *Scedosporium apiospermum*. A 10-month-old female Maremmano-Abruzzese sheepdog showing weakness, lethargy, lateral decubitus, miosis and muscular rigidity was presented. Rodenticide poisoning was clinically suspected for the differential diagnosis. However, postmortem examinations revealed the presence of a swollen and soft subcutaneous nodule located near the right inguinal breast, which was associated with massive enlargement of the inguinal lymph nodes and small disseminated, cream-colored nodules in the kidneys and mesentery. Multiple fungal pyogranulomas were observed upon histological examination. Fungal isolation from the kidneys, breast and inguinal lymph nodes was performed. The internal transcribed spacer (ITS) sequences from the fungal colony DNA were searched in BLAST in the NCBI GenBank for species identification. The sequences of the fungi isolated from the kidney and breast cultures showed 100% sequence identity with sequences from *Scedosporium apiospermum*.

**Conclusions:**

This report shows that *Scedosporium apiospermum* may act as a primary pathogen in young and apparently healthy dogs and represents an important pathogen that should be considered during the diagnostic process, particularly when a fungal infection is suspected.

## Background

Fungi belonging to the *Scedosporium* genus are saprophytic and ubiquitous with a worldwide distribution and result in a wide range of clinical signs and disease presentations both in humans and in animals [1, 2]. There has been a steady increase in the infection rates of this species in both immunocompromised and immunocompetent hosts, which acts as an opportunistic or primary pathogen. In addition, the high degree of intrinsic antifungal resistance makes these infections difficult to manage [2–4].

In humans, *Scedosporium* spp. can infect different tissues and organs, including skin and soft tissues, muscle, joint and bone, in cases of localized infection. Systemic and often fatal disseminated infections involve the central nervous system (CNS), myocardium, lungs and other splanchnic organs, especially in immunocompromised hosts [2, 5].

Few cases of scedosporiosis have been reported in animals and have mainly been of traumatic origin, such as surgery, but the real prevalence is probably underestimated due to a lack of awareness [1]. Scedosporiosis in dogs has often associated with localized infections (i.e. nasal infection, eumycetoma, or keratomycosis) [1, 3, 6–10], but in rare cases, it has been associated with disseminated infections [11].

The taxonomy of *Scedosporium* spp. has undergone considerable change over the last decade following the introduction of molecular phylogenetics, which led to an increase in resolution at and below the species level. Currently, the *Scedosporium* genus includes ten species, and among these, *Scedosporium apiospermum* is considered one of the most pathogenic [2, 5].

The clinical and histopathological features of *Scedosporium apiospermum* infections are very similar to those of *Aspergillus* spp. and *Fusarium* spp. infections, making the diagnosis challenging [3, 12]. Sporulation in culture is required for fungal identification, but even confusion with other morphologically similar species is possible, and in most cases, the etiological agent is identified by molecular methods [12, 13].

This case report describes the clinical and pathological features and the diagnostic process of a rare systemic and fatal infection caused by *Scedosporium apiospermum* in a Maremmano-Abruzzese sheepdog. To the best of our knowledge, this is the first reported case of its kind in Italy.

## Case presentation

In late October 2019, a 10-month-old female Maremmano-Abruzzese sheepdog weighing approximately 35 kg was presented to the “Clinica Veterinaria Guardiese”, Guardiagrele, Abruzzo, Italy, with weakness, lethargy, lateral decubitus, miosis and muscular rigidity. This dog had lived indoors starting from the age of 2 months. Deworming and vaccination were regularly conducted. Four days before the first clinical signs, the dog was moved to the garden around the house; its health status was excellent, and on a daily basis, it was taken for a walk in the countryside by the owner. An episode of diarrhea, vomiting and anorexia was reported 24–36 h before referral to the veterinary clinic.

Physical examination revealed a rectal temperature of 39 °C, a pulse rate of 110 beats/min and a respiratory rate of 30 breaths/min. The mucous membranes were normal, and the capillary refill time was less than 2 s. No abnormal lung or heart sounds were noted. The right inguinal mammary gland and the surrounding subcutaneous tissues were moderately swollen. Routine hematobiochemical examination indicated mild-moderate leukocytosis (20,500 cells/ml; reference interval: 6000–17,000 cells/ml) with mild neutrophilia (16,200 cells/ml; reference interval: 3000–10,000 cells/ml). Total serum protein, total serum alkaline phosphatase activity, alanine aminotransferase activity, urea, creatinine and glucose concentration were within the reference intervals. No abnormalities were observed in the radiographic and ultrasound examinations.

Supportive therapy was immediately established with intravenous fluids (Ringer’s lactate solution, 10 ml/kg/hour) and antibiotics (amoxicillin-clavulanic acid, 12.5 mg/kg). After a few hours, the clinical conditions worsened precipitously, and the dog died. Rodenticide poisoning was clinically suspected, so the carcass was immediately submitted for necropsy to the “Istituto Zooprofilattico Sperimentale dell’Abruzzo e del Molise” (IZSAM), Teramo, Italy.

During the postmortem examination, a 5 cm-diameter swollen and soft subcutaneous nodule was found in the right inguinal area adjacent to the breast. The inguinal lymph nodes were markedly enlarged and edematous, and both kidneys had a roughened subcapsular surface. In addition, cream-colored lardaceous nodules measuring 1–2 mm in diameter were observed throughout the mesentery and in the renal cortex. Other organs were grossly normal.

Tissue samples (inguinal lymph nodes, breast and adjacent nodules, liver, kidney, spleen, lung, mesentery, brain and spinal cord) were fixed in 10% neutral buffered formalin and embedded in paraffin. All samples were routinely processed for histology and stained with hematoxylin and eosin (HE). Selected samples presenting gross and microscopic lesions (kidney, breast, inguinal lymph nodes and mesentery) were also stained with periodic acid-Schiff (PAS) and Grocott stain. The aforementioned tissues, including stomach content, were also collected and frozen at − 20 °C for toxicological and microbiological analysis.

All tissues and stomach content proved to be negative for rodenticide poisons based on toxicological examinations.

**Fig. 1:**
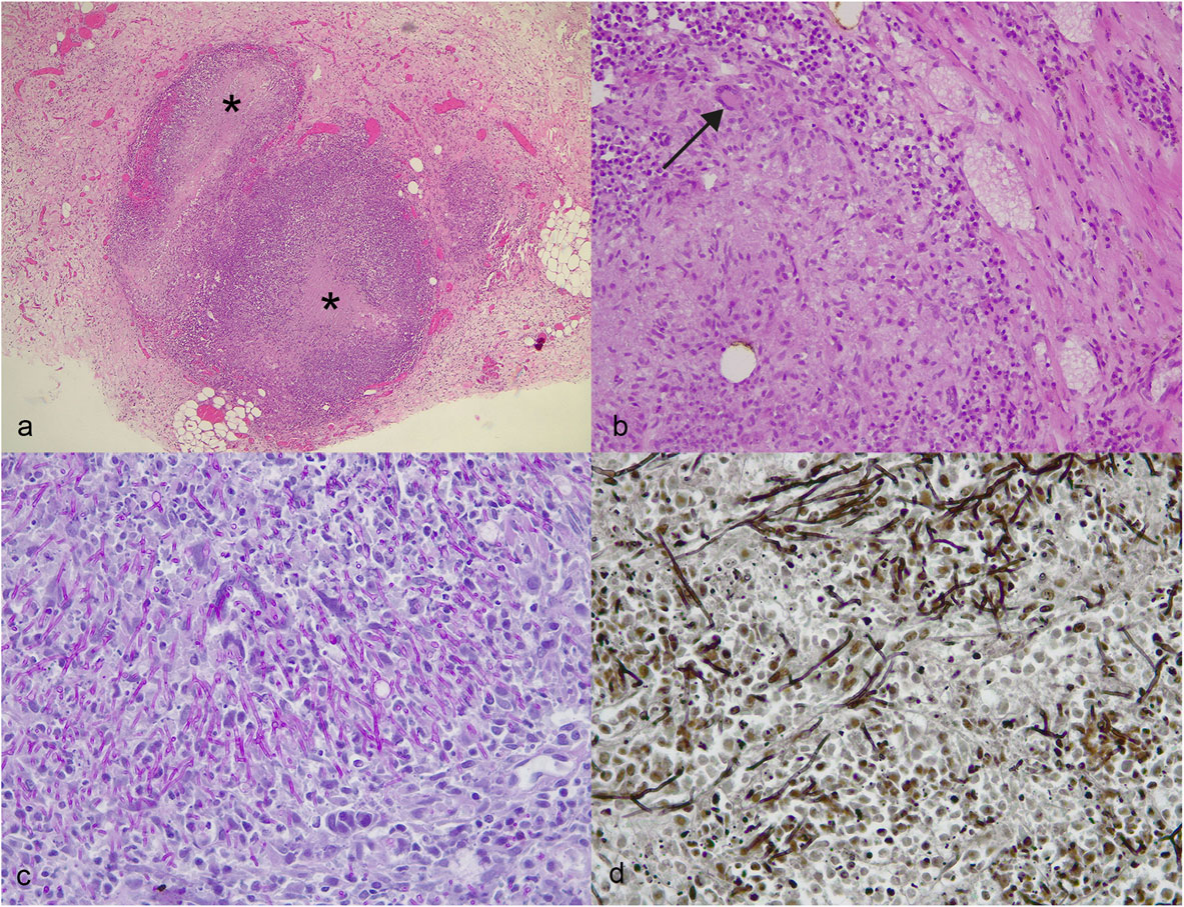
Inguinal subcutaneous tissue, dog. **a** Two pyogranulomas with a “target-like” appearance are visible in the deep subcutis near the mammary gland. The centers of the granulomas are necrotic (black asterisks). The peripheral zones are filled with irregularly arranged macrophages and lymphocytes. Hematoxylin and Eosin (HE) staining. **b** A multinucleated giant cell (black arrow) is observed near the necrotic core of the pyogranuloma. HE staining. **c** Periodic acid-Schiff staining showed PAS-positive structures with parallel walls and dichotomous branching. **d** Irregular fungal black hyphae are visible. Grocott staining

Histological examination of the kidney, mesentery, lymph nodes and breast revealed numerous foci of dense inflammatory infiltrates with a “target-like” appearance, which consisted of a central core of necrosis and cellular debris in association with a moderate number of neutrophils and epithelioid macrophages surrounded by a zone of irregularly arranged macrophages and lymphocytes (Fig. 1a). Multinucleated Langhans-type giant cells were occasionally observed (Fig. 1b). No microscopic lesions were recorded in the brain, spinal cord, liver, lung or spleen. PAS staining revealed a hyaline structure with parallel walls that exhibited dichotomous branching (Fig. 1c). Grocott staining also showed abundant irregular black-colored fungal hyphae (Fig. 1d). Fungal hyphae were also observed in blood vessels of the mammary gland and kidneys. The histopathological findings were consistent with severe multifocal fungal pyogranulomas. Based on only the hyphae histological morphology, an interim diagnosis of disseminated aspergillosis was made, with definitive diagnosis of the agent pending the culture results.

**Fig. 2:**
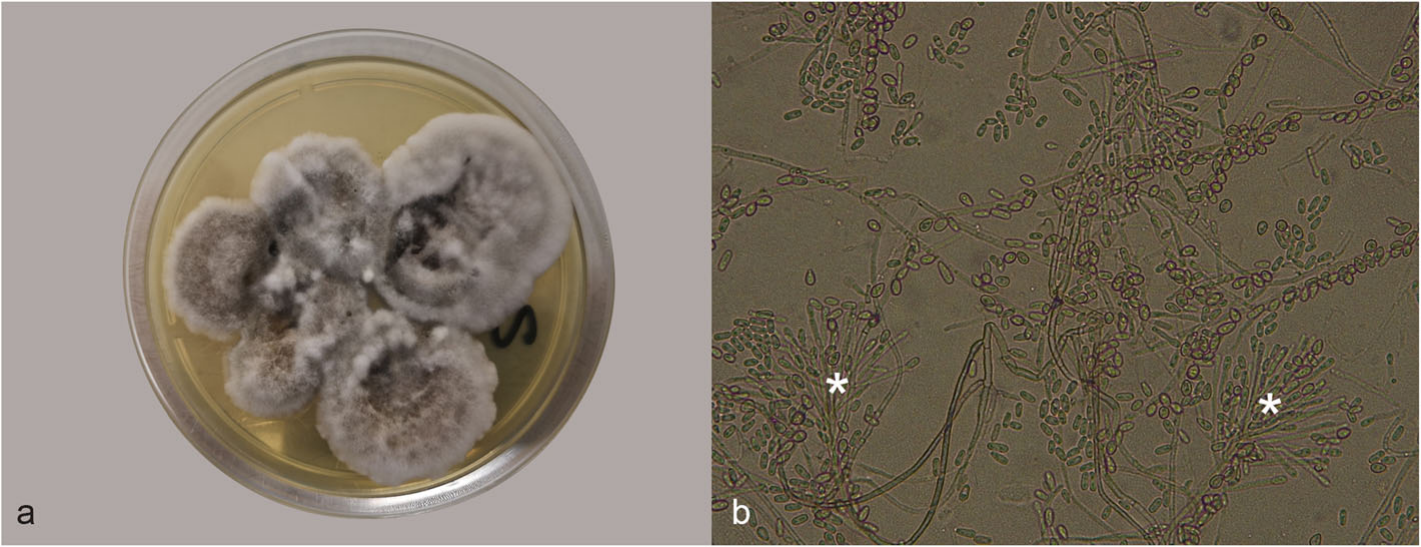
Culture of *Scedosporium apiospermum* from the kidney on Sabouraud Dextrose Agar (SDA). **a** Gray–white cottony and wooly colonies with brownish centers and irregular margins were observed after 7 days of culture. **b** Light microscopy showed the conidial structures of *Scedosporium apiospermum*; the conidiophores contained conidiogenous cells producing conidia. The apical part of a synnema with conidia is also visible (white asterisks)

Frozen samples from kidney, inguinal lymph nodes, breast, liver, spleen, brain and spinal cord were collected for mycological examination. Briefly, all tissues were homogenized and inoculated onto Sabouraud Dextrose Agar (SDA), and the plates were incubated at 30 °C. Fungal growth was initially observed after 72–96 h as white or gray colonies. After 5–7 days of culture, the colonies became dark-gray or grayish-brown in the center and gray-white in the peripheral zone and were woolly to cottony in texture (Fig. 2a). On the reverse side of the plates, the color of the colonies was dark with brownish-black zones in the center. Brain, spinal cord, spleen and liver samples tested negative for fungal isolation. Microscope slides prepared through the tape strip technique (without staining) revealed the characteristic conidial structures of *Scedosporium apiospermum*. Microscopic examination showed long tapering conidiophores that were both simple and branched (synnema). Conidiophores gave rise to ovoid or clavate conidia (Fig. 2b). The fungal morphology was consistent with that of fungi belonging to the *Scedosporium* genus. Biomolecular methods were used to accurately identify the species. In detail, fungal hyphae grown on SDA plates were collected (1 cm^2^) with sterile handling for DNA extraction. Colonies from kidney and breast tissues were processed by the Maxwell 16 Tissue DNA Purification kit with the Maxwell 16 Instrument according to the manufacturer’s instructions (Promega, Madison, US). A 570-bp region of the internal transcribed spacer (ITS) in the nuclear ribosomal DNA of both samples was amplified with the ITS5 and ITS4 primers as previously described [14]. The PCR products were then purified using the GeneAll ExpinTMPCR Kit (GeneAll) and sent to Eurofins Genomics Srl for sequencing. The raw ITS sequences were assembled using SeqScape v2.5 (Applied Biosystems, Foster City, CA) and subjected to a BLAST search in the NCBI GenBank for species identification. The fungi sequences isolated from both the kidney and breast cultures showed 100% sequence identity with sequences from *Scedosporium apiospermum* (accession number: KY122843). The sequences were deposited in GenBank (accession number: MT135178, MT137381).

## Discussion and conclusions

A small number of systemic scedosporiosis caused by *Scedosporium apiospermum* have been reported in canine species [13, 15–17] and the involvement of the urinary tract and reproductive apparatus have been rarely reported [18–20]. Interestingly, many disseminated infections in dogs have had no apparent route of entry or predisposing factors [13], and as reported in the present case, the route of infection remains unknown. Speculatively, it is possible that the dog contracted *Scedosporium apiospermum* infection through a skin abrasion in proximity to the inguinal region, causing mycetoma and fungal granulomatous mastitis visible in the postmortem examination as a swollen inguinal nodule near the right inguinal breast. Then, after the invasion of the mammary gland and subcutis, the fungi migrated into the regional lymph nodes and spread throughout the lymphatic and blood vessels in the kidney and mesentery.

It is well known that human *Scedosporium* infection occurs both in immunocompetent and immunocompromised patients [2]. The predisposing factors that lead to systemic infection are still obscure. As demonstrated in murine models, the route of infection and presence of other pathologies and/or therapy could affect the severity and outcome of the infection [1, 11, 12, 21]. In dogs, a breed-specific predisposition toward infection has been reported only in the German Shepherd, which is probably due to abnormal cell-mediated immunity and/or immunoglobulin A regulation and function [17, 22]. It is important to remark that in our case, the dog did not show any anamnestic, clinical, radiographic, ultrasound or hematological evidence of concurrent disease, and no specific predisposition of Maremmano-Abruzzese sheep dogs has been described so far. Therefore, as reported in humans, *Scedosporium apiospermum* could also act as a primary pathogen in young and apparently healthy dogs [2, 3].

Fungi belonging to the genus *Scedosporium* are recognized to have special tropism for the CNS during disseminated infection, particularly in immunocompromised human hosts [21]. However, in our case, the brain and the spinal cord did not show any pathological changes, and the isolation of fungi from these tissues was unsuccessful. It has been hypothesized that CNS infection may appear as a manifestation of systemic disease in the absence of a clear focus of spread [2]. Similarly, the neurological signs (such as muscular rigidity and miosis) observed in this case were not triggered by fungal invasion of the CNS but rather were likely caused by the comatose state of the dog.

The diagnosis of *Scedosporium apiospermum* infection is often time-consuming, challenging and complicated by the numerous clinical and morphologic similarities to other fungal granulomatous diseases, such as aspergillosis and fusariomycosis [3, 9]. Notably, the systemic form described herein was initially diagnosed as poisoning by clinicians; this interim diagnosis was deduced only because of the acute progression of the clinical signs and the presence of sudden neurological signs, as no poison bait was found by the owner, and was finally excluded by toxicological examinations. Then, at the postmortem examination, a neoplastic disease was considered in the differential diagnosis, while the presumption of systemic mycosis (initially hypothesized to be caused by aspergillosis due to the morphology of the hyphae) was possible only on the basis of histological and histochemical (PAS and Grocott) findings, which are of crucial importance in such cases [1]. Only after careful morphological examination of the colonies was a diagnosis of scedosporiosis possible. For a definitive diagnosis and species identification, we used nucleotide sequence-based analysis, which represents the current gold standard for fungal identification [2].

Treatment of *Scedosporium* infections remains challenging because of the intrinsically limited susceptibility of these fungi to all currently used antifungal drugs [1]. In this case, it was not possible to treat the dog with antifungal drugs, and after isolation, an in vitro antifungal susceptibility test was not performed. It would be of great interest to evaluate the sensitivity of *Scedosporium* species to the drugs commonly used in veterinary practice. Indeed, in most clinical cases and especially in systemic cases with invasion of SNC, the affected hosts did not survive [1, 12].

Although the true prevalence of scedosporiosis in veterinary medicine seems to be lower than that in human medicine, most cases may remain undiagnosed or be misdiagnosed as aspergillosis [1].

In conclusion, *Scedosporium apiospermum* represents an important emerging pathogen to be considered during the diagnostic process both in humans and in animals, particularly when a fungal infection is suspected.

## Data Availability

The datasets used and/or analyzed during the current study are available from the corresponding author on reasonable request.

## Abbreviations

CNS: Central nervous system
HE: Hematoxylin and Eosin
PAS: Periodic acid-Schiff
SDA: Sabouraud Dextrose Agar
ITS: Internal transcribed spacer
